# Effect of Annealing Time on Corrosion Behaviours of Zr_56_Cu_19_Ni_11_Al_9_Nb_5_ in Hank Solution

**DOI:** 10.3390/ma18051132

**Published:** 2025-03-03

**Authors:** Zhiying Zhang, Jianling Zhou, Kun Wang, Jinguo Gao, Qinyi Zhang, Xinlei Jiang, Chenhao Yu, Zikai Zhou, Haonan Liu

**Affiliations:** 1 School of Materials Science and Engineering, Wuhan University of Technology, Wuhan 430070, China; zjl331217@whut.edu.cn (J.Z.); zhqy@whut.edu.cn (Q.Z.); jxl_330972@whut.edu.cn (X.J.); 345048@whut.edu.cn (C.Y.); 345091@whut.edu.cn (Z.Z.); 344878@whut.edu.cn (H.L.); 2 School of Energy and Power Engineering, Huazhong University of Science and Technology, Wuhan 430074, China; hust_wk@hust.edu.cn; 3 Hubei Hongle Cable Co., Ltd., Honghu, Jingzhou 433225, China; jinguo.gao@hbhongle.com

**Keywords:** metallic glass, annealing, Hank solution, corrosion, electrochemical properties

## Abstract

The microstructures of the as-cast and annealed Zr_56_Cu_19_Ni_11_Al_9_Nb_5_ were investigated by X-ray diffraction (XRD) and scanning electron microscopy (SEM), their microhardness values were tested, and their corrosion behaviours in Hank solution were studied. XRD results and SEM analysis showed that the as-cast sample was amorphous, and crystallisation occurred in the samples annealed at 923 K for 5–30 min with crystals of Zr_2_Cu and Zr_2_Ni. Microhardness gradually increased and then levelled off, due to higher crystallisation degree with longer annealing time. Passivation occurred for all the samples in Hank solution. Prolonged annealing time leads to the initial rise and then a drop in corrosion resistance. Annealing for 5 min resulted in the highest corrosion resistance, with high corrosion potential E_corr_ at −0.007 V_SCE_, versus saturated calomel electrode (SCE), i.e., 0.234 V_SHE_, versus standard hydrogen electrode (SHE), the smallest corrosion current density i_corr_ at 2.20 × 10^−7^ A·cm^−2^, the highest pitting potential E_pit_ at 0.415 V_SCE_ (i.e., 0.656 V_SHE_), the largest passivation region E_pit_–E_corr_ at 0.421 V_SHE_, the largest arc radius, and the largest sum of charge transfer resistance and film resistance R_ct_ + R_f_ at 15489 Ω·cm^2^. Annealing for 30 min led to the lowest corrosion resistance, with low E_corr_ at −0.069 V_SCE_ (i.e., 0.172 V_SHE_), large i_corr_ at 1.32 × 10^−6^ A·cm^−2^, low E_pit_ at −0.001 V_SCE_ (i.e., 0.240 V_SHE_), small E_pit_ − E_corr_ at 0.068 V_SHE_, the smallest arc radius, and the smallest R_ct_ + R_f_ at 4070 Ω·cm^2^. When the annealing time was appropriate, the homogeneous microstructure of nanocrystals in an amorphous matrix resulted in improved passivation film, leading to the rise of corrosion resistance. However, if the annealing time was prolonged, the inhomogeneous microstructure of larger crystals in an amorphous matrix resulted in a drop in corrosion resistance. Localised corrosion was observed, with corrosion products of ZrO_2_, Cu_2_O, CuO, Ni(OH)_2_, Al_2_O_3_, and Nb_2_O_5_.

## 1. Introduction

Bulk metallic glasses (BMGs) and bulk metallic glass composites (BMGCs) have been widely studied [1,2,3]. Zr-based BMGs showed promising biomedical applications due to high strength and hardness, good corrosion resistance, good glass forming ability, good biocompatibility, good wear resistance, and low elastic modulus [4,5]. Zr_61_Cu_17.5_Ni_10_Al_7.5_Si_4_ exhibited good corrosion resistance in Hank solution, and good biocompatibility, indicating good potential for biomedical applications [6]. Corrosion resistance is affected by chemical composition. Proper addition of Nb and Ti led to better corrosion resistance [7,8]. Zr_55_Al_20_Co_25−x_Nb_x_ (x = 0, 2.5, 5) displayed good corrosion resistance in Hank solution and phosphate-buffered saline (PBS) solution, and the increase in Nb content led to better corrosion resistance and reduced water contact angle [7]. Zr_65−x_Ti_x_Cu_20_Al_10_Fe_5_ (x = 4, 6) showed better corrosion resistance than Zr_65−x_Ti_x_Cu_20_Al_10_Fe_5_ (x = 0, 2) in PBS solution [8]. Zr_60+x_Ti_2.5_Al_10_Fe_12.5−x_Cu_10_Ag_5_ (x = 0, 2.5, 5) exhibited good corrosion resistance in PBS solution, and higher Zr content resulted in better corrosion resistance due to higher Zr/Al ratio in the passive film [9]. Zr_46_(Cu_4.5/5.5_Ag_1/5.5_)_46_Al_8_ showed better biocompatibility and better corrosion resistance than Zr_51.9_Cu_23.3_Ni_10.5_Al_14.3_ and Zr_51_Cu_25_Ni_10_Al_9_Ti_5_ in Hank solution due to the amorphous structure and the formation of Al_2_O_3-_enriched passive film [10]. Zr_53_Al_16_Co_26_Pt_5_ showed the best corrosion resistance, followed by Zr_53_Al_16_Co_26_Pd_5_, and Zr_53_Al_16_Co_26_Au_5_ showed the worst corrosion resistance in PBS solution [11]. Zr_55.8_Al_19.4_(Co_1−x_Cu_x_)_24.8_ (x = 0–0.8) were prepared and Zr_55.8_Al_19.4_(Co_1−x_Cu_x_)_24.8_ (x = 0.3) illustrated good corrosion resistance in PBS solution, the largest 12 mm casting diameter, and good mechanical properties [12]. Zr_60.5_Hf_3_Al_9_Fe_4.5_Cu_23_ and Zr_58.6_Al_15.4_Co_18.2_Cu_7.8_ illustrated good corrosion resistance in PBS solution, and good antibacterial properties [13,14]. Zr_40_Ti_37_Co_12_Ni_11_, Zr_50_Ti_32_Cu_13_Ag_5_, Zr_46_Ti_40_Ag_14_ and Zr_46_Ti_43_Al_11_ displayed better corrosion resistance than Ti and 316L steel in PBS solution, and Zr_46_Ti_40_Ag_14_ showed better antibacterial properties than Zr_46_Ti_43_Al_11_ [15,16]. Zr_45_Ti_36_Fe_11_Al_8_ exhibited better corrosion resistance than Ti in PBS solution due to the amorphous structure and the stable ZrO_2_ and TiO_2_ film [17]. Compared with Zr_65_Cu_18_Al_10_Ni_7_, Zr_55_Cu_30_Al_10_Ni_5_ displayed higher microhardness, higher E_corr_ and smaller E_pit_ − E_corr_ in PBS solution, and better wear resistance both in air and in PBS solution [18]. Zr_62_Cu_22_Al_10_Fe_5_Dy_1_ showed amorphous structure, good corrosion resistance in PBS solution, artificial saliva solution (ASS), Hank solution, and artificial blood plasma (ABP) solution, as well as good biocompatibility [19]. Zr_37_Co_34_Cu_20_Ti_9_ displayed good corrosion resistance in PBS, ASS, ABP, and Hank solutions, and good biocompatibility [20].

The corrosion behaviours of Zr-based BMGs were influenced by annealing temperature and time. The as-cast Zr_56_Co_28_Al_16_ sample and the samples annealed at 660 K (<T_g_), 800 K and 973 K (>T_x_) for 5 h displayed poorer corrosion resistance in Ringer’s solution and poorer biocompatibility with higher annealing temperature [21]. Zr_58_Nb_3_Cu_16_Ni_13_Al_10_ samples were annealed at 523 K and 673 K (<T_g_), as well as at 773 K and 873 K (>T_x_), for 6 h, and with higher annealing temperature, the microhardness gradually increased, and the corrosion resistance in 1 mol/L H_2_SO_4_ solution at 333 K gradually decreased [22]. Zr_60_Cu_20_Ni_8_Al_7_Hf_3_Ti_2_ samples were annealed at 500 K and 600 K (<T_g_) and remained amorphous, and with higher annealing temperature, the microhardness rose, and the corrosion resistance in 0.01 mol/L H_2_SO_4_ solution slightly dropped [23]. Zr_50.7_Ni_28_Cu_9_Al_12.3_ samples were annealed at 719 K (T_g_~T_x_), 768 K (>T_x_), and 810 K for 30 min, and the structures were amorphous, nanocrystals of ZrO_2_ in an amorphous matrix, and crystals of ZrO_2_, Al_2_Zr, Cu_10_Zr_7_, and CuZr_2_ in an amorphous matrix, respectively, and the sample annealed at 768 K exhibited the highest corrosion resistance in 0.5 mol/L H_2_SO_4_, 1 mol/L NaCl and 1 mol/L HCl solutions [24]. Zr_41.2_Cu_12.5_Ni_10_Ti_13.8_Be_22.5_ and Zr_57_Cu_15.4_Ni_12.6_Al_10_Nb_5_ samples annealed at 0.9T_g_ for 4 h showed better corrosion resistance in NaCl solution than the as-cast sample, due to the decreased free volume [25]. Zr_68_Al_8_Ni_8_Cu_16_ samples annealed at 673 K and 713 K displayed crystals of Zr_2_Ni and Zr_2_Cu in the amorphous matrix with a crystallinity of 10% and 77%, and the sample annealed at 713 K exhibited the maximum microhardness, the lowest strength, the smallest plasticity, and the worst corrosion resistance in 1 mol/L HCl solution [26]. The Zr_65_Cu_15_Ni_12.5_Al_7.5_ samples were annealed at 643 K, 663 K and 683 K (T_g_~T_x_) for 10 min, and with higher annealing temperature, the plasticity gradually decreased, and the corrosion resistance in 3.5% NaCl solution gradually decreased, and the cast sample showed higher corrosion resistance than the annealed samples [27]. The fully crystallised Zr_48_Cu_46.5_Al_4_Nb_1.5_ sample obtained by flash-annealing showed better corrosion resistance than the as-cast Zr_48_Cu_46.5_Al_4_Nb_1.5_ sample and Zr_47.5_Cu_47.5_Al_5_ sample in 3.5% NaCl solution and 0.05 mol/L H_2_SO_4_ solution [28]. Zr_56_Cu_19_Ni_11_Al_9_Nb_5_ as-cast sample and samples annealed at 623 K (<T_g_), 723 K (T_g_~T_x_), 823 K and 923 K (>T_x_) for 30 min displayed good corrosion resistance in PBS solution, and at higher annealing temperature, the corrosion resistance firstly rose and then dropped, with the best corrosion resistance for the sample annealed at 823 K and the worst corrosion resistance for the sample annealed at 923 K [29].

Zr_60_Cu_20_Al_10_Fe_5_Ti_5_ as-cast sample and the samples annealed at 648 K (slightly < T_g_) for 1 min and 5 min showed amorphous structure and the sample annealed at 873 K (>T_x_) for 1 min displayed crystalline structure with crystals of Zr_2_Cu and Al_2_Zr_3_. They exhibited gradually increased hardness, higher E_corr_ and better corrosion resistance in Hank solution, due to structural relaxation, reduced free volume and increased crystallisation [30]. The Zr_65_Cu_17.5_Fe_10_Al_7.5_ samples were annealed at 573 K (<T_g_) for 0.5–4 h, and with prolonged annealing time, the microhardness firstly rose and then dropped, and the sample annealed at 573 K for 1 h showed the maximum microhardness of 487 HV, the highest plasticity of 7.1%, and the highest corrosion resistance in 3.5% NaCl solution due to the decreased free volume [31]. Zr_35_Ti_30_Be_26.75_Cu_8.25_ samples were annealed at 683 K (T_g_~T_x_) for 5 min and 12 min, and the corrosion resistance in 3.5% NaCl solution decreased, and the corrosion resistance increased after holding at 77 K for 30 min, and decreased after cryogenic cycling [32]. The as-cast Zr_50.7_Cu_28_Ni_9_Al_12.3_ sample and the samples annealed at 736 K (T_g_~T_x_) for 140, 164, and 193 min displayed amorphous structure and crystals in the amorphous matrix with the volume fraction of the crystalline phases of 14%, 40%, and 100%, and the sample annealed for 140 min exhibited the best corrosion resistance in the simulated groundwater, and with longer annealing time, the corrosion resistance gradually dropped [33]. The as-cast Zr_59_Ti_6_Cu_17.5_Fe_10_Al_7.5_ sample and the samples annealed at 573 K (<T_g_) for 0.5–2 h displayed amorphous structure, and the sample annealed at 573 K for 4 h exhibited crystals of Al_3_Zr_2_ in amorphous matrix, and with prolonged annealing time, the strength and plasticity firstly rose and then dropped, with the highest strength and the largest plasticity for the sample annealed at 573 K for 0.5 h, and the corrosion resistance of all the samples remained good in PBS solution [34].

The effects of microstructure, annealing temperature and time displayed complex effects on the corrosion resistance of Zr-based BMGs and BMGCs in simulated bio-environments, and further study is necessary. In this work, the microstructures of as-cast Zr_56_Cu_19_Ni_11_Al_9_Nb_5_ metallic glass and the samples annealed at 923 K (>T_x_) for 5–30 min were studied by X-ray diffraction (XRD) and scanning electron microscopy (SEM), their microhardness values were tested, and their corrosion behaviours in Hank solution were studied. Our work helps the further understanding of corrosion mechanisms of Zr-based metallic glass and composites and further research and development in biomedical applications.

## 2. Materials and Methods

### 2.1. Material Preparation

**Figure 1:**
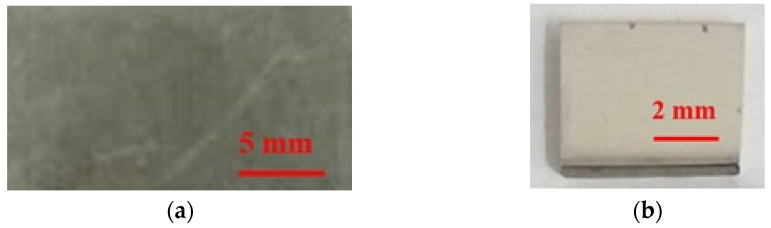
Zr_56_Cu_19_Ni_11_Al_9_Nb_5_ samples, (**a**) as-cast sample, (**b**) polished sample.

**Figure 2:**
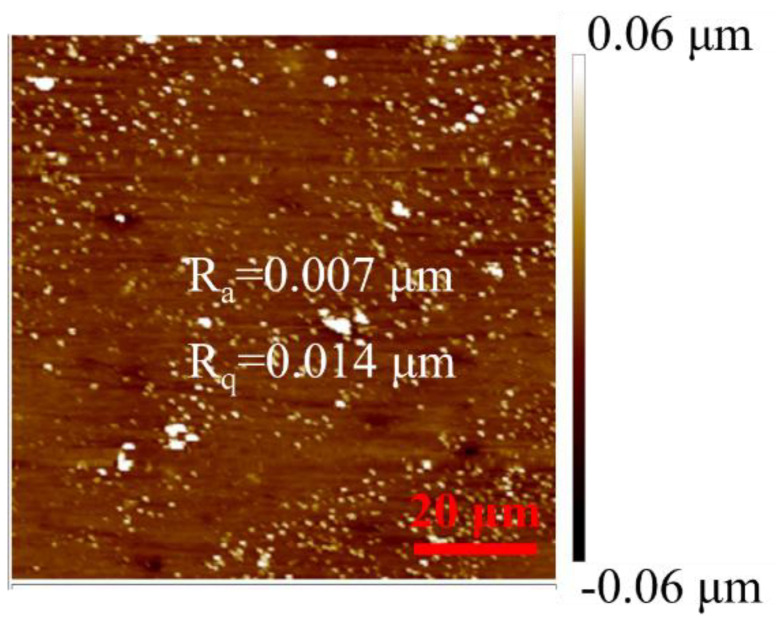
Surface roughness of Zr_56_Cu_19_Ni_11_Al_9_Nb_5_ sample.

Zr_56_Cu_19_Ni_11_Al_9_Nb_5_ samples were obtained through arc-melting of pure Zr, Cu, Ni, Al and Nb [29]. The samples were cut to 5 mm × 4 mm × 1 mm, as shown in Figure 1, and were annealed at 923 K (far above T_x_) for 5, 8, 10, 15, and 30 min. The surfaces were ground using 800–1500 grit sandpaper, polished using 2.5 and 0.5 µm diamond paste, and then cleaned in acetone. The surface roughness of the sample was determined by Dimension IconIR atomic force microscopy (Bruker, MA, USA), as shown in Figure 2, with R_a_ around 0.007 μm and R_q_ around 0.014 μm.

### 2.2. Tests

XRD analysis was performed using a D8 Advance X-ray diffractor (Bruker, Karlsruhe, Germany) with a diffraction angle 2θ in the range of 20–90°, and an SEM image was obtained using Zeiss Ultra Plus field emission scanning electron microscopy (Zeiss, Jena, Germany) with the voltage of 5 kV, in order to investigate the microstructures of the as-cast sample and the samples annealed at 923 K for 5–30 min. The amorphous-to-crystalline ratio is equal to the area of the amorphous structure divided by the area of the crystalline structure. Vickers microhardness measurements were carried out using a MICRO-586 microhardness tester (Shandong Shancai Testing Instrument Co., Ltd., Yantai, Shandong, China) with a load of 2 N and a holding time of 10 s, and the measurements were repeated 10 times for each sample. Potentiodynamic polarisation (PP) tests and electrochemical impedance spectroscopy (EIS) tests of the as-cast and annealed samples in Hank solution were carried out through the CHI 660E electrochemical station (Shanghai CH Instruments, Shanghai, China) [29]. The composition of Hank solution is as follows, 0.14 g/L CaCl_2_, 0.1 g/L MgCl_2_·6H_2_O, 0.1 g/L MgSO_4_·7H_2_O, 0.35 g/L NaHCO_3_, 1 g/L D-glucose, 0.01 g/L phenol red. The saturated calomel electrode (SCE) was used as the reference electrode, and the graphite electrode was used as the counter electrode. The electrodes were stabilised in Hank solution at open circuit potential (OCP) for 60 min. In PP tests, the potential was scanned from −0.8 V_SCE_ (i.e., −0.559 V_SHE_) to 0.5 V_SCE_ (i.e., 0.741 V_SHE_) at 0.33 mV/s. In EIS tests, the complex impedance was obtained at OCP, with the frequency of 10^−2^–10^5^ Hz and the amplitude of 5 mV. Zsimpwin 3.6 software was used to fit the EIS data through the appropriate equivalent circuit (EC). After PP and EIS tests, the corrosion morphology and products were analysed by SH11/YF-III optical microscopy (OM, Shanghai Optical Instrument Factory, Shanghai, China), Zeiss Ultra Plus field emission SEM (Zeiss, Jena, Germany), X-Max 50 X energy dispersive X-ray spectroscopy (EDS, Oxford Instruments, Abingdon, UK) and Thermo Scientific ESCALAB 250Xi X-ray photoelectron spectroscopy (XPS, Thermofisher Scientific, Waltham, MA, USA) [29]. The measurement accuracy of the equipment is shown in Table 1.

## 3. Results and Discussion

### 3.1. Microstructure Analysis

**Figure 3:**
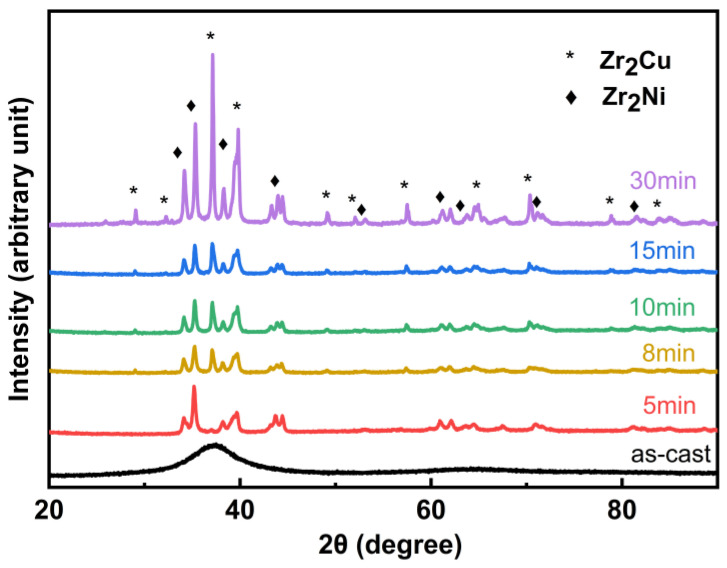
XRD patterns of Zr_56_Cu_19_Ni_11_Al_9_Nb_5_ as-cast sample and samples annealed at 923 K for 5–30 min.

**Figure 4:**
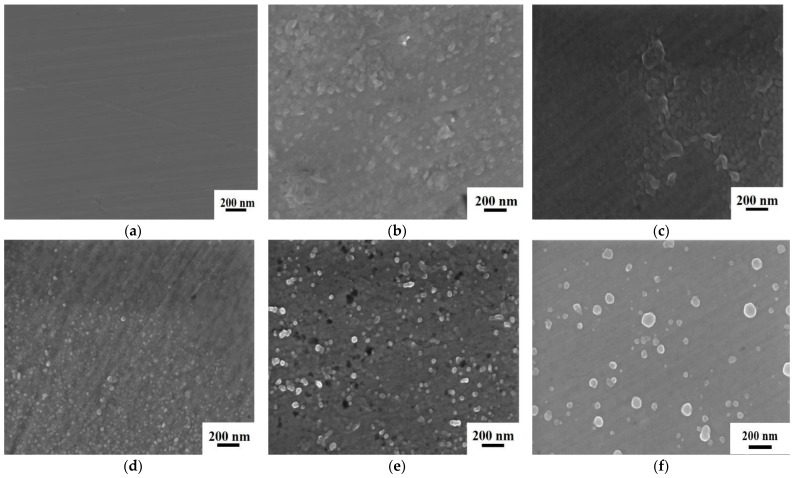
SEM images of Zr_56_Cu_19_Ni_11_Al_9_Nb_5_ as-cast sample, and samples annealed at 923 K for 5–30 min. (**a**) as-cast (**b**) 5 min (**c**) 8 min (**d**) 10 min (**e**) 15 min (**f**) 30 min.

The XRD patterns and SEM images of the as-cast Zr_56_Cu_19_Ni_11_Al_9_Nb_5_ sample and the samples annealed at 923 K for 5–30 min are shown in Figure 3 and Figure 4. The as-cast sample shows an amorphous structure, and crystallisation occurred in the samples annealed at 923 K for 5–30 min with crystals of Zr_2_Cu and Zr_2_Ni. With longer annealing time, the intensity of the diffraction peaks gradually increased, and the crystallisation degree gradually increased, with crystallisation percentages of 0, 17%, 18%, 27%, 27% and 85% for the as-cast sample and the samples annealed at 923 K for 5 min, 8 min, 10 min, 15 min and 30 min, respectively. SEM images display the amorphous structure of the as-cast sample and microstructures of nanocrystals in the amorphous matrix of the annealed samples. With prolonged annealing time, the number of crystals firstly increased and then decreased, and the size of crystals gradually increased, due to the increase in crystallisation degree and the growth and coalescence of crystals. The amorphous-to-crystalline ratio was 4.9, 4.6, 2.7, 2.7 and 0.2 for the samples annealed at 923 K for 5 min, 8 min, 10 min, 15 min and 30 min, respectively.

### 3.2. Microhardness

**Figure 5:**
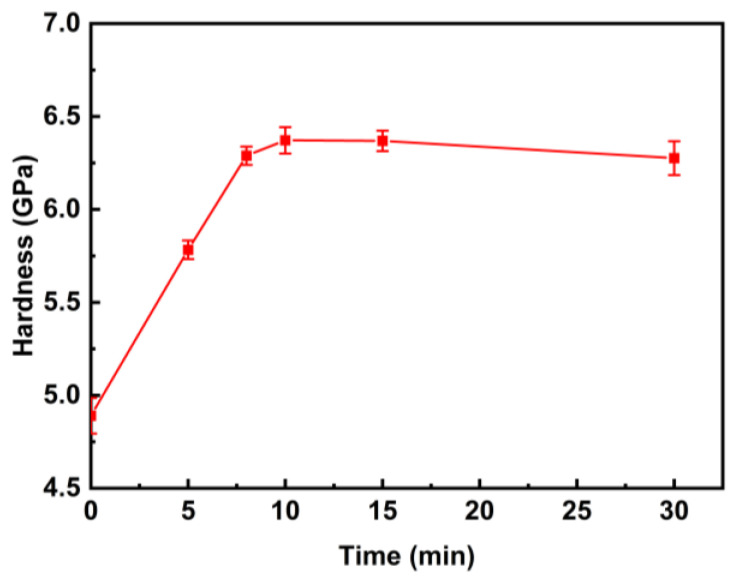
Microhardness of Zr_56_Cu_19_Ni_11_Al_9_Nb_5_ as-cast sample and samples annealed at 923 K for 5–30 min.

Figure 5 shows the microhardness of the as-cast Zr_56_Cu_19_Ni_11_Al_9_Nb_5_ sample and the samples annealed at 923 K for 5–30 min. With a longer annealing time, the microhardness gradually rose from 4.89 GPa (i.e., 499 Hv) to 6.37 GPa (i.e., 650 Hv) and then levelled off. The brittleness gradually increased, reaching the maximum, and then slightly dropped. It is due to the increase in crystallisation degree with prolonged annealing time, as shown by XRD analysis and SEM images. The interface between the crystals and the amorphous matrix and the grain boundaries hindered the deformation, leading to the increase in microhardness. A similar trend was reported for Zr_65_Cu_17.5_Fe_10_Al_7.5_ samples annealed at 573 K (<T_g_) for 0.5–4 h, and the microhardness increased from 422 Hv for the as-cast sample to 487 Hv for the sample annealed at 573 K for 1 h and then dropped to 444 Hv for the sample annealed at 573 K for 4 h [31].

### 3.3. Corrosion Behaviours

**Figure 6:**
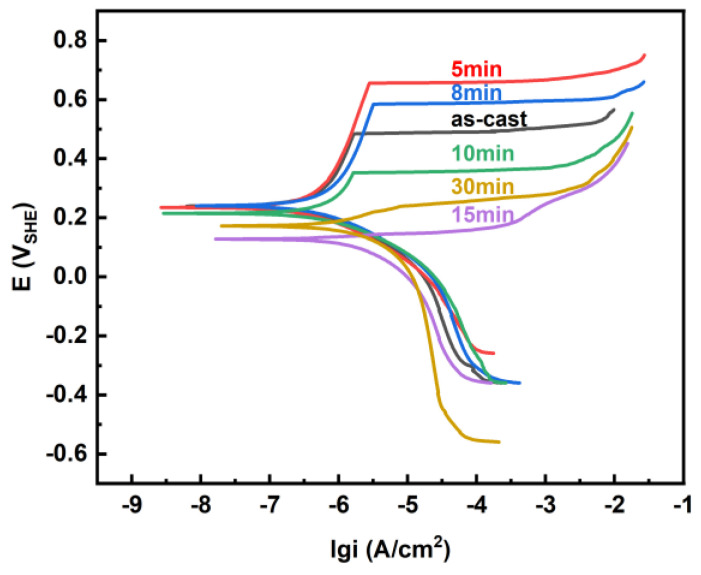
PP curves of Zr_56_Cu_19_Ni_11_Al_9_Nb_5_ as-cast sample and samples annealed at 923 K for 5–30 min in Hank solution.

**Figure 7:**
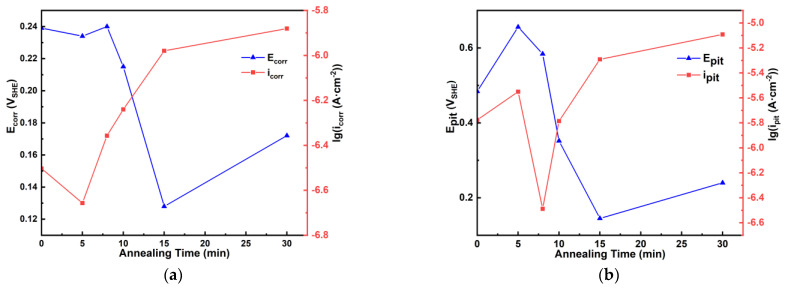
Change of E_corr_, i_corr_, E_pit_, i_pit_ with annealing time for as-cast sample and samples annealed at 923 K for 5–30 min. (**a**) change of E_corr_ and i_corr_ with annealing time, (**b**) change of E_pit_ and i_pit_ with annealing time.

**Figure 8:**
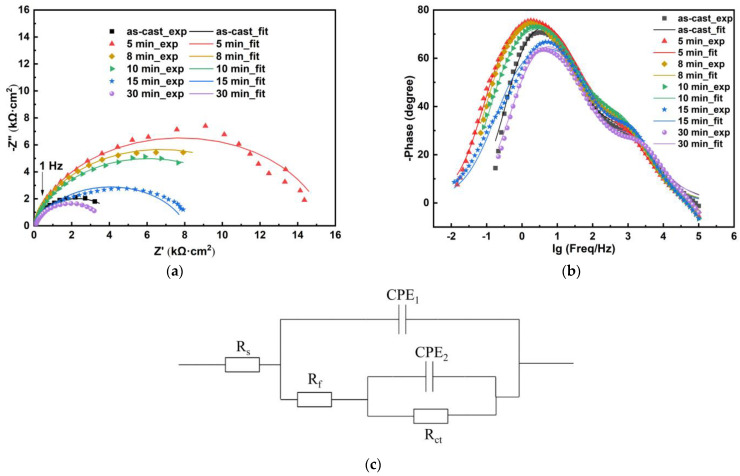
The EIS results for Zr_56_Cu_19_Ni_11_Al_9_Nb_5_ as-cast sample and samples annealed at 923 K for 5–30 min in Hank solution, (**a**) Nyquist plots, (**b**) Bode plots, (**c**) the equivalent circuit diagram.

Figure 6 shows the PP curves for the as-cast Zr_56_Cu_19_Ni_11_Al_9_Nb_5_ sample and the samples annealed at 923 K for 5–30 min in Hank solution, and Table 2 summarises the obtained electrochemical parameters. Figure 7 illustrates the change of corrosion potential E_corr_, corrosion current density i_corr_, pitting potential E_pit_, pitting current density i_pit_ with annealing time. For the as-cast sample and the samples annealed at 923 K for 5–10 min, E_pit_ was determined from the kink where the current density increased sharply. For the samples annealed at 923 K for 15 and 30 min, E_pit_ was determined from the intersection point of the tangent lines where the current density increased dramatically. Passivation occurred for all the samples, indicating good corrosion resistance in Hank solution. With longer annealing time, E_corr_ and E_pit_ first rose and then dropped, i_corr_ and i_pit_ first dropped and then rose, and the corrosion resistance first rose and then dropped. The sample annealed at 923 K for 5 min exhibited the best corrosion resistance, with high E_corr_ at −0.007 V_SCE_ (i.e., 0.234 V_SHE_), the smallest i_corr_ at 2.20 × 10^−7^ A·cm^−2^, the highest E_pit_ at 0.415 V_SCE_ (i.e., 0.656 V_SHE_), and the largest passivation region E_pit_ − E_corr_ at 0.421 V_SHE_. The sample annealed at 923 K for 30 min exhibited the worst corrosion resistance, with low E_corr_ at −0.069 V_SCE_ (i.e., 0.172 V_SHE_), large i_corr_ at 1.32 × 10^−6^ A·cm^−2^, low E_pit_ at −0.001 V_SCE_ (i.e., 0.240 V_SHE_) and small E_pit_ − E_corr_ at 0.068 V_SHE_. Figure 8 illustrates the Nyquist plots, Bode plots and the equivalent circuit diagram for the EIS results of the as-cast Zr_56_Cu_19_Ni_11_Al_9_Nb_5_ sample and the samples annealed at 923 K for 5–30 min in Hank solution, and Table 3 summarises the obtained electrochemical parameters.

**Figure 9:**
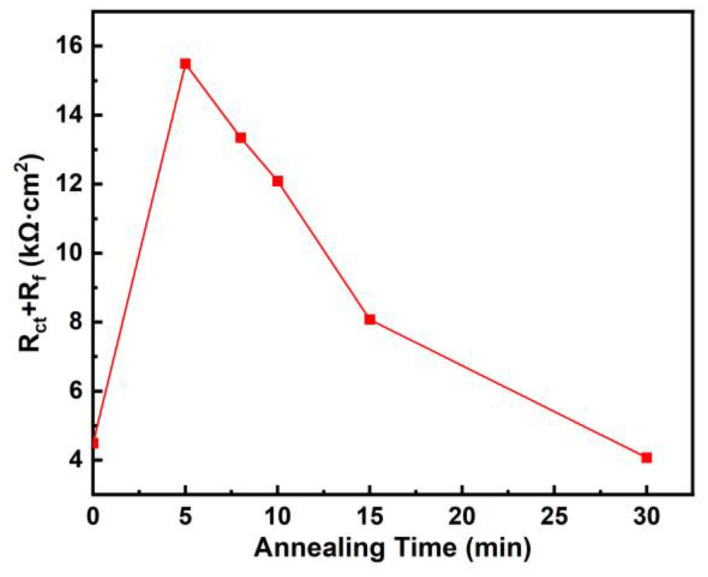
Change of R_ct_ + R_f_ with annealing time for Zr_56_Cu_19_Ni_11_Al_9_Nb_5_ as-cast sample and samples annealed at 923 K for 5–30 min.

The Nyquist plots displayed a half-circle, indicating that the control step was the electrochemical reaction accompanied by the transfer of electrons. The Bode plots showed two time constants, with frequencies around 3 Hz and 1000 Hz. The maximum phase was reached around 3 Hz. The equivalent circuit diagram as shown in Figure 8c was previously used for the fitting of EIS results of Zr_45_Ti_36_Fe_11_Al_8_ in PBS solution [16], Zr_58_Nb_3_Cu_16_Ni_13_Al_10_ as-cast sample and samples annealed at 523 K, 673 K, 773 K and 873 K in 1 mol/L H_2_SO_4_ solution at 333 K [21], Zr_52_Cu_32_Al_10_Ni_6_ in 0.05–0.5 mol/L NaF solutions [35], Al_86_Ni_9_Y_5_ as-spun sample and the samples annealed at 423 K for 5 min and 30 min, at 543 K for 5 min, and at 673 K for 5 min in 0.6 mol/L NaCl [36]. The passivation film contains defects. R_s_ represents the solution resistance between the working electrode and the reference electrode. R_f_ represents the film resistance, and R_ct_ represents the charge transfer resistance at the interface between the solution and the film. CPE_1_ and CPE_2_ represent the constant phase element (CPE) of the film and the capacitance of the double layer between the solution and the film. The impedance $Z={Y_{0}}^{-1}\omega^{-n}\left[\cos\left(\frac{n\pi }{2}\right)-j\sin\left(\frac{n\pi }{2}\right)\right]$. Y_0_ is the constant, ω is the angular frequency, and n is the parameter between 0 and 1. Figure 9 shows the change of the sum of charge transfer resistance and film resistance R_ct_ + R_f_ with annealing time. With the increase in annealing time, the arc radius gradually increased and then decreased, R_ct_ + R_f_ gradually decreased and then increased, indicating that the corrosion resistance in Hank solution first increased and then decreased. The sample annealed at 923 K for 5 min showed the largest arc radius and the largest R_ct_ + R_f_, 15,489 Ω·cm^2^, indicating the best corrosion resistance. The sample annealed at 923 K for 30 min displayed the smallest arc radius and the smallest R_ct_ + R_f_, 4070 Ω·cm^2^, indicating the worst corrosion resistance.

**Figure 10:**
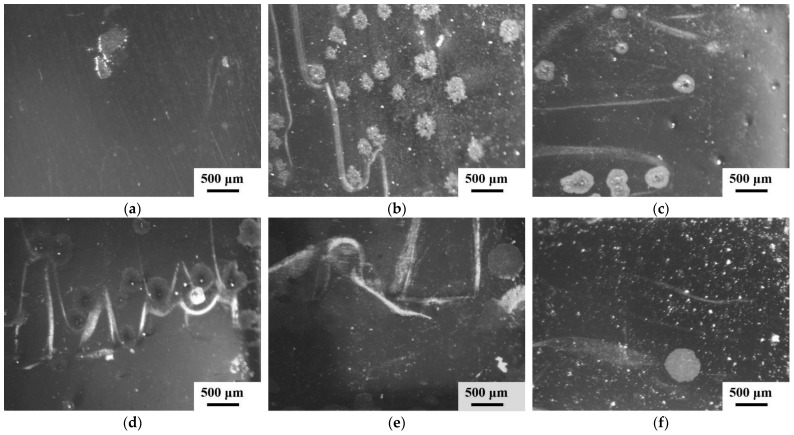
OM images of the as-cast and annealed Zr_56_Cu_19_Ni_11_Al_9_Nb_5_ samples after PP tests. (**a**) as-cast (**b**) 5 min (**c**) 8 min (**d**) 10 min (**e**) 15 min (**f**) 30 min.

**Figure 11:**
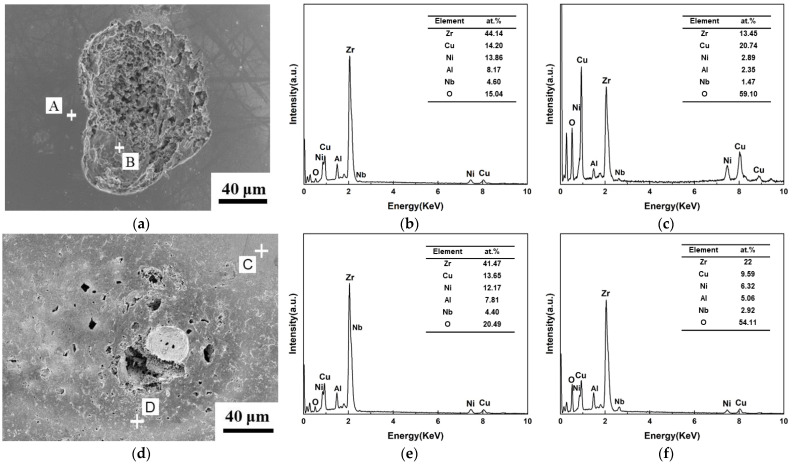

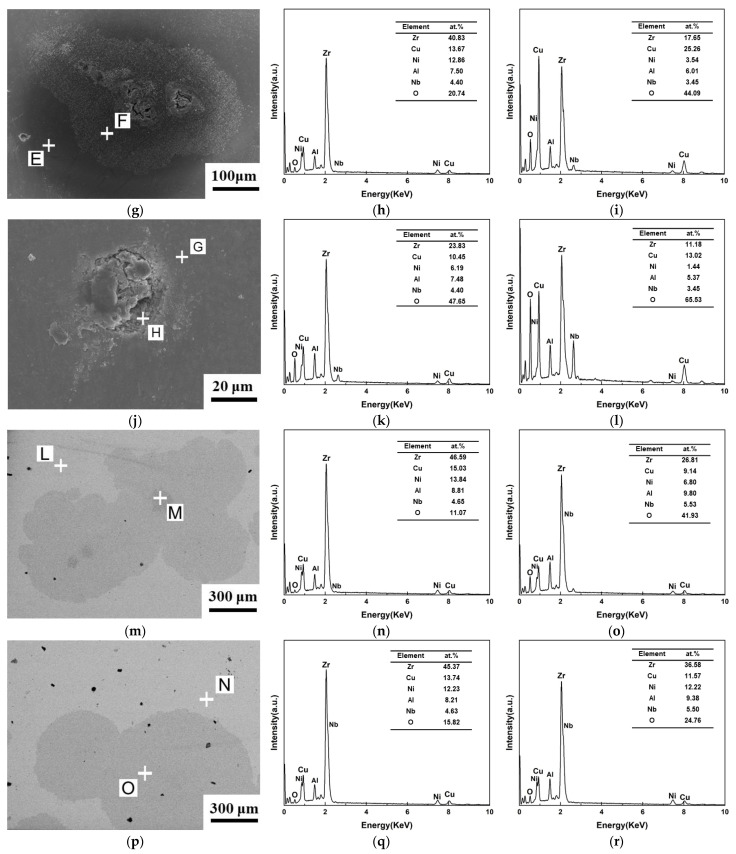
SEM images and EDS analysis of the as-cast and annealed Zr_56_Cu_19_Ni_11_Al_9_Nb_5_ samples after PP tests. (**a**) SEM, as-cast (**b**) EDS, spot A (**c**) EDS, spot B (**d**) SEM, 5 min (**e**) EDS, spot C (**f**) EDS, spot D (**g**) SEM, 8 min (**h**) EDS, spot E (**i**) EDS, spot F (**j**) SEM, 10 min (**k**) EDS, spot G (**l**) EDS, spot H (**m**) SEM, 15 min (**n**) EDS, spot L (**o**) EDS, spot M (**p**) SEM, 30 min (**q**) EDS, spot N (**r**) EDS, spot O.

Figure 10 and Figure 11 exhibit OM images, SEM images and EDS analysis of the as-cast Zr_56_Cu_19_Ni_11_Al_9_Nb_5_ sample and the samples annealed at 923 K for 5–30 min after PP tests in Hank solution. Pitting was observed in all the samples. Spots A, C, E, G, L and N are located in the non-corroded area, with a low oxygen content of 11–48 at.%, and concentrations of Zr, Cu, Ni, Al and Nb of 24–47 at.%, 10–15 at.%, 6–14 at.%, 7–9 at.%, 4–5 at.%, which are similar to the original 56 at.%, 19 at.%, 11 at.%, 9 at.%, and 5 at.%, respectively. Spots B, D, F, M and O are located in the corroded area, with a higher oxygen content of 25–66 at.% and concentrations of Zr, Cu, Ni, Al and Nb of 10–37 at.%, 9–25 at.%, 1–12 at.%, 2–10 at.%, 1–6 at.%, respectively, much lower than the original concentrations. The decrease in Zr content is the largest.

The as-cast Zr_56_Cu_19_Ni_11_Al_9_Nb_5_ sample exhibited an amorphous structure, resulting in good corrosion resistance in Hank solution. The annealed sample at 923 K (>T_x_) for 5 min displayed a homogeneous microstructure of nanocrystals of Zr_2_Cu and Zr_2_Ni in the amorphous matrix, as confirmed by XRD analysis and SEM image. The improved stability of the passivation film led to higher corrosion resistance. With a longer annealing time, the crystallisation content rose. The microstructure became inhomogeneous, with larger crystals of Zr_2_Cu and Zr_2_Ni in the amorphous matrix, leading to a drop in corrosion resistance.

**Figure 12:**
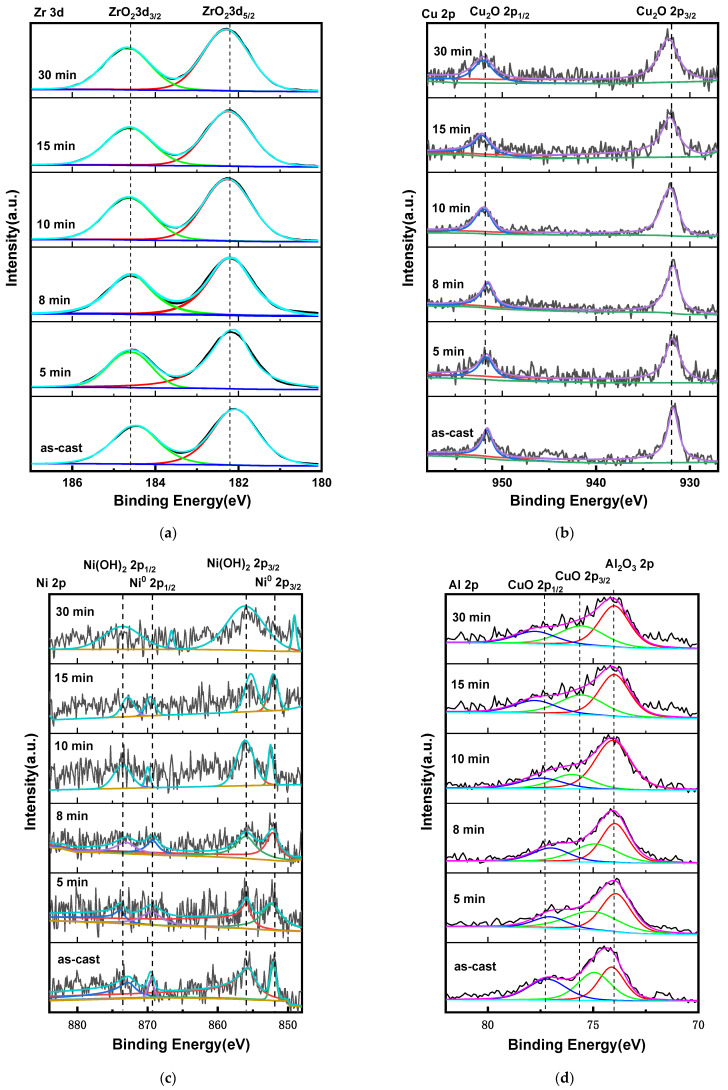

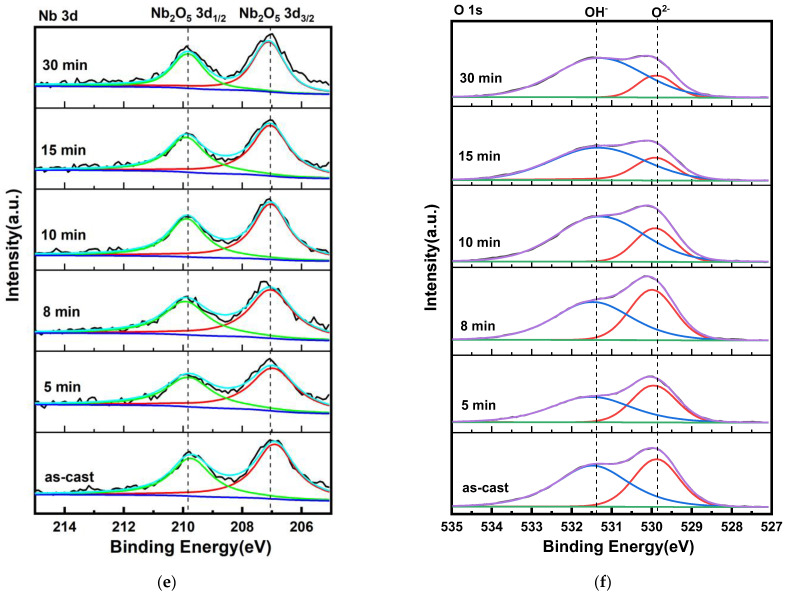
XPS analysis of the as-cast and annealed Zr_56_Cu_19_Ni_11_Al_9_Nb_5_ samples after EIS tests. (**a**) Zr 3d (**b**) Cu 2p (**c**) Ni 2p (**d**) Al 2p and Cu 2p (**e**) Nb 3d (**f**) O 1s.

Figure 12 shows the XPS analysis of the Zr_56_Cu_19_Ni_11_Al_9_Nb_5_ as-cast sample and the samples annealed at 923 K for 5–30 min after EIS tests in Hank solution. The experimental data, the base line, the fitted peaks and the sum of fitted peaks were illustrated in different colours. The peaks at 182.2 eV and 184.6 eV exhibited Zr^4+^ 3d_5/2_ and Zr^4+^ 3d_3/2_ [3,29,37]. The peaks at 932.0 eV and 952.0 eV represented Cu^+^ 2p_3/2_ and Cu^+^ 2p_1/2_ [3,29,37]. The peaks at 75.6 eV and 77.6 eV indicated Cu^2+^ 2p_3/2_ and Cu^2+^ 2p_1/2_ [3,29,37]. The peaks at 851.9 eV and 856.0 eV indicated Ni^0^ 2p_3/2_ and Ni^2+^ 2p_3/2_, and the peaks at 869.5 eV and 873.5 eV indicated Ni^0^ 2p_1/2_ and Ni^2+^ 2p_1/2_ [3,29]. The peak at 74.0 eV represented Al^3+^ 2p [29,37]. The peaks at 207.0 eV and 209.8 eV indicated Nb^5+^ 3d_3/2_ and Nb^5+^ 3d_1/2_ [3,29]. The peaks at 529.8 eV and 531.3 eV represented O^2−^ and OH^−^ [3,29,37]. Therefore, the corrosion products consist of ZrO_2_, CuO, Cu_2_O, Ni(OH)_2_, Al_2_O_3,_ and Nb_2_O_5_.

The electrode potential of Al/Al^3+^, Zr/Zr^4+^, Nb/Nb^5+^, Ni/Ni^2+^, Cu/Cu^2+^, and Cu/Cu^+^ is as follows, −1.662 V_SHE_, −1.529 V_SHE_, −1.200 V_SHE_, −0.250 V_SHE_, 0.337 V_VSH_, and 0.521 V_SHE_, respectively. Al, Zr, and Nb are more active than Ni and Cu, due to lower potential, and the following corrosion reactions occur first. 2Al + 3H_2_O → Al_2_O_3_ + 3H_2_, Zr + 2H_2_O → ZrO_2_ + 2H_2_, 2Nb + 5H_2_O → Nb_2_O_5_ + 5H_2_. The corrosion of Ni and Cu occurs next, with higher potential. Ni + 2H_2_O → Ni(OH)_2_ + H_2_, Cu + H_2_O → CuO + H_2_, 2Cu + H_2_O → Cu_2_O + H_2_.

Larsson et al. reported that for the Zr_59.3_Cu_28.8_Al_10.4_Nb_1.5_ sample manufactured by selective laser melting, the ion release under both simulated physiological and inflammatory conditions is as follows, Zr > Al > Cu > Nb due to the lower potential of Zr and Al, and the ion release under inflammatory condition is higher than that under simulated physiological condition [38]. Zr is a biocompatible element, and Al and Cu are trace elements present in the human body. Ni is a toxic element, which may cause an allergy response. Large ion release higher than the legal limits may cause health problems [39,40,41].

## 4. Conclusions

The as-cast Zr_56_Cu_19_Ni_11_Al_9_Nb_5_ sample showed an amorphous structure, and the samples annealed at 923 K for 5–30 min showed crystals of Zr_2_Cu and Zr_2_Ni in the amorphous matrix. With prolonged annealing time, the crystallisation degree gradually increased, and the microhardness gradually increased and then levelled off. Passivation was observed for all the samples in Hank solution, indicating good corrosion resistance. With a longer annealing time, the corrosion resistance first rose and then dropped. The annealed sample at 923 K for 5 min displayed the highest corrosion resistance, with high E_corr_ at −0.007 V_SCE_ (i.e., 0.234 V_SHE_), the smallest i_corr_ at 2.20 × 10^−7^ A·cm^−2^, the highest E_pit_ at 0.415 V_SCE_ (i.e., 0.656 V_SHE_), the largest E_pit_ − E_corr_ at 0.421 V_SHE_, the largest arc radius, and the largest R_ct_ + R_f_ at 15,489 Ω·cm^2^. The annealed sample at 923 K for 30 min exhibited the lowest corrosion resistance, with low E_corr_ at −0.069 V_SCE_ (i.e., 0.172 V_SHE_), large i_corr_ at 1.32 × 10^−6^ A·cm^−2^ (i.e., 500% increase), low E_pit_ at −0.001 V_SCE_ (i.e., 0.240 V_SHE_), small E_pit_ − E_corr_ at 0.068 V_SHE_ (i.e., 84% decrease), the smallest arc radius, and the smallest R_ct_ + R_f_ at 4070 Ω·cm^2^ (i.e., 74% decrease). When the annealing time was appropriate, the microstructure was homogeneous with nanocrystals in an amorphous matrix, resulting in improved passivation film and the rise of corrosion resistance. However, if the annealing time was prolonged, the microstructure became inhomogeneous, with larger crystals in an amorphous matrix, leading to a drop in corrosion resistance. Localised corrosion was observed, with corrosion products of ZrO_2_, Cu_2_O, CuO, Ni(OH)_2_, Al_2_O_3_, and Nb_2_O_5_. Zr_56_Cu_19_Ni_11_Al_9_Nb_5_ sample annealed at 923 K for 5 min exhibited potential candidate for biomedical applications due to high hardness and good corrosion resistance in Hank solution. In the future, in vivo tests can be performed to investigate the biocompatibility.

## Figures and Tables

**Table 1:** List of equipment and the measurement accuracy.

Equipment	Model	Parameter	Accuracy
X-ray diffractor	D8 Advance	angle	10^−2^°
Electrochemical workstation	CHI 660E	potential	10^−4^ V
Electrochemical workstation	CHI 660E	current	10^−8^ A
Microhardness tester	MICRO-586	microhardness	10^−1^ MPa

**Table 2:** PP results of Zr_56_Cu_19_Ni_11_Al_9_Nb_5_ as-cast sample and samples annealed at 923 K for 5–30 min in Hank solution.

Annealing Time (min)	E_corr_ (V_SHE_)	i_corr_ (A·cm^−2^)	E_pit_ (V_SHE_)	i_pit_ (A/cm^2^)	E_pit_ − E_corr_ (V_SHE_)
0	0.239	3.14 × 10^−7^	0.484	1.67 × 10^−6^	0.245
5	0.234	2.20 × 10^−7^	0.656	2.81 × 10^−6^	0.421
8	0.240	4.40 × 10^−7^	0.584	3.25 × 10^−7^	0.344
10	0.215	5.75 × 10^−7^	0.352	1.64 × 10^−6^	0.138
15	0.128	1.05 × 10^−6^	0.145	5.11 × 10^−6^	0.017
30	0.172	1.32 × 10^−6^	0.240	8.09 × 10^−6^	0.068

**Table 3:** EIS analysis results for as-cast and annealed samples in Hank solution.

Annealing Time (min)	EIS Fitting Results							
	R_s_ (Ω·cm^2^)	Y_01_ (Ω^−1^·s^n^·/cm^2^)	n_1_	R_f_ (Ω·cm^2^)	Y_02_ (Ω^−1^·s^n^·/cm^2^)	n_2_	R_ct_ (Ω·cm^2^)	R_ct_ + R_f_ (Ω·cm^2^)
0	11	1.05 × 10^−4^	0.94	4444	2.68 × 10^−4^	0.64	46	4490
5	11	1.19 × 10^−4^	0.89	15,460	1.92 × 10^−4^	0.71	29	15,489
8	11	1.19 × 10^−4^	0.90	13,300	1.55 × 10^−4^	0.72	44	13,344
10	12	1.02 × 10^−4^	0.88	12,050	1.21 × 10^−4^	0.75	37	12,087
15	16	1.15 × 10^−4^	0.79	8053	2.60 × 10^−5^	0.89	23	8076
30	17	9.17 × 10^−5^	0.69	47	9.24 × 10^−5^	0.87	4023	4070

## Data Availability

The data shown in this study are available on request from the corresponding author. The data are not publicly available due to privacy reasons.

## Abbreviations

Nomenclature	
ABP	artificial blood plasma solution
ASS	artificial saliva solution
BMGs	Bulk metallic glasses
BMGCs	bulk metallic glass composites
CPE	constant phase element
EC	equivalent circuit
EDS	energy dispersive X-ray spectroscopy
EIS	electrochemical impedance spectroscopy
OCP	open circuit potential
PBS	phosphate-buffered saline
PP	Potentiodynamic polarisation
XPS	X-ray photoelectron spectroscopy
SCE	Saturated calomel electrode
SEM	scanning electron microscopy
XRD	X-ray diffraction
E_corr_	corrosion potential
E_pit_	pitting potential
E_pit_ − E_corr_	passivation region
i_pit_	pitting current density
i_corr_	corrosion current density
R_ct_	charge transfer resistance
R_f_	film resistance
R_s_	the solution resistance between the working electrode and the reference electrode
ω	angular frequency
